# Wet Grip Performance Evaluation Method of All-Steel Radial Tires Based on Braking Force Coefficient

**DOI:** 10.3390/polym17202726

**Published:** 2025-10-10

**Authors:** Shengzhong Long, Juqiao Su, Gege Huang, Youshan Wang, Jian Wu

**Affiliations:** 1 National Key Laboratory of Science and Technology on Advanced Composites in Special Environments, Harbin Institute of Technology, Harbin 150090, China; longshengzhong@outlook.com (S.L.); wangys@hit.edu.cn (Y.W.); 2 Guizhou Tyre Co., Ltd., Guiyang 550008, China; sujuqiao@gtc.com.cn; 3 Center for Rubber Composite Materials and Structures, Harbin Institute of Technology, Weihai 264209, China

**Keywords:** tire, wet grip performance, braking force coefficient, tread rubber

## Abstract

Tires are composed of various rubber polymers and reinforcing carcasses, and their wet skid resistance is influenced by the coupled effects of multiple factors. The braking force coefficient (BFC) is the primary performance indicator for evaluating tire wet skid resistance. This study proposes a novel method for evaluating the BFC of tires by integrating laboratory-simulated wet road tests with finite element simulations. A 295/60R22.5 all-steel radial tire was selected as the test object, and the simulation results showed good agreement with the experimental data, with a BFC error of 7.14%. This consistency confirms the reliability and accuracy of the proposed model in predicting tire wet grip performance. This study also investigated the effects of different working conditions of the tested tire on the BFC. The results showed that the wet grip performance of the tire on wet concrete surfaces was significantly lower than that on wet asphalt surfaces. Specifically, the BFC increased with the increase in braking slip ratio, decreased slightly with the rise in tire inflation pressure, and exhibited relatively low sensitivity to vertical load variations. All these results demonstrate that this integrated evaluation method provides targeted guidance for the mechanical performance optimization of tire tread rubber composites.

## 1. Introduction

Wet skid resistance, rolling resistance, and wear resistance represent the three primary performance metrics of tires, collectively termed the “Magic Triangle” of tread compound properties [1]. As the primary contact interface between the tire and road surface, the tread compound is most susceptible to dynamic loading effects and consequently exerts the dominant influence on the wet skid resistance performance of tires.

The factors influencing the wet skid resistance of tread compounds are highly complex. In addition to tread pattern, actual road conditions, and water film thickness [2], the inherent properties of the tread compound itself and the type of filler used play critical roles. For traditional carbon black-filled tire tread compounds, wet skid performance is closely correlated with dynamic hysteresis loss at low temperatures. Consequently, the loss factor tan δ at 0 °C is commonly employed as an indirect indicator of the material’s wet skid resistance [3]. Y. Saito [4] demonstrated a strong linear correlation between the loss factor tan δ at low temperatures/high frequencies and the wet skid resistance of rubber compounds. Wang et al. [5] reported that in single-filler reinforced rubber systems, wet skid resistance exhibits a strong correlation with dynamic viscoelastic properties. However, this correlation becomes significantly weaker in rubber compounds incorporating multiple fillers of distinct characteristics. Sae et al. [6] conducted a comprehensive study on the partial replacement of carbon black with silica. Their results demonstrated that as the silica content increased, both the wet skid resistance and rolling resistance of the tires improved significantly. However, the wear resistance was compromised compared to carbon black-filled compounds.

Researchers have undertaken extensive studies on tire braking efficiency. Nahmias et al. [7] conducted tire braking tests to establish the correlation between the viscoelastic properties of tread compounds and wet friction coefficients. Mauro et al. [8] executed coupled friction tests across five distinct textured test tracks, establishing a correlation between the Wet Grip Index (WGI) and the International Friction Index (IFI). Kane et al. [9] introduced a tool designed to estimate tire–road friction, which was utilized in braking tests conducted at varying speeds on wet textured roads. Löwer et al. [10,11] developed a physical model to describe the interaction between rubber and a rough surface with water acting as an intermediate medium, taking into account factors such as surface macro-roughness, water height, tire pattern, and vehicle speed on braking performance. Ma Bin et al. [12] integrated pavement fractal properties with the viscoelastic behavior of tread rubber to create an enhanced model for the sliding friction coefficient, derived through simplified viscoelastic analysis. Zhao et al. [13] performed block-on-pavement friction tests to examine how water film thickness on asphalt pavement influences tire–road friction coefficients. Jiao et al. [14] developed a novel viscoelastic friction belt in the laboratory and systematically investigated the correlation between water film thickness and tire wet skid resistance by progressively increasing the water film thickness. Their findings revealed that water film thickness significantly influences tire motion patterns and slip rates. During braking, tires were more prone to skidding on the road surface when the water film was either excessively thick (>3 mm) or extremely thin (approximately 0–1 mm).

Simulation methods offer enhanced flexibility for analyzing the various factors that influence tire wet traction and provide theoretical insights into the mechanisms of tire–road interaction. The implicit time integration finite element algorithm is most commonly utilized for contact analysis [15]. Browne et al. [16] proposed a theoretical model to assess tire wet skid resistance. Cho et al. [17] adopted a velocity discretization approach during braking, applying the law of energy conservation to evaluate vehicle stopping distances on both dry and wet surfaces. Jeong et al. [18] developed a method to estimate pavement friction coefficients under conditions of thin water film, addressing the computational challenges associated with simulating thin-film hydroplaning. Fwa et al. [19] advanced the modeling of wet pavement by creating a three-dimensional numerical tire–pavement–water film model, analyzing how velocity affects wet skid resistance across varying water film thicknesses. Löwer et al. [20] constructed a finite element model of a single tread block to investigate the pattern-dependent wet braking behavior of passenger car tires on asphalt, elucidating the superior performance of the sipe pattern. Y.S. Wang [21] developed an aircraft tire hydroplaning model for smooth runways using TYABAS V3.0 software version in conjunction with the CEL algorithm, conducting a qualitative analysis of tire force characteristics under different operating conditions and emphasizing the impact of tire inflation pressure on hydroplaning. Wang Guolin et al. [22] effectively employed the CEL algorithm within ABAQUS to simulate fluid–structure interaction problems and evaluate tire anti-skid performance on wet pavement. Zhang et al. [23] created a three-dimensional rough road model utilizing the harmonic superposition method, simulating the sealing effect of water films on wet roads as a pseudo-hydrodynamic bearing. They applied a friction model to analyze the wet skid resistance of a 205/55R16 tire featuring two different tread patterns.

As eco-friendly ”green tires” are being developed in the tire industry, the use of dynamic viscoelasticity to characterize the wet skid resistance mechanism of carbon black- and silica-reinforced rubber compounds faces significant challenges [24]. This study presents an innovative approach that integrates indoor rubber friction testing with simulation techniques, adhering to evaluation criteria from official testing standards. Focusing on a 295/60R22.5 all-steel radial tire as the test subject, this method merges the precision of controlled laboratory experiments with the efficiency and adaptability of computational simulations. It effectively addresses the spatial and environmental constraints of traditional testing environments, enabling rapid and straightforward measurement of the BFC on wet surfaces. Furthermore, this approach explores the impacts of road surface, slip ratio, inflation pressure, and load on the BFC, yielding valuable insights to enhance tire wet grip performance.

## 2. Experimental and Simulation Methodology

### 2.1. Braking Force Coefficient (BFC)

**Figure 1:**
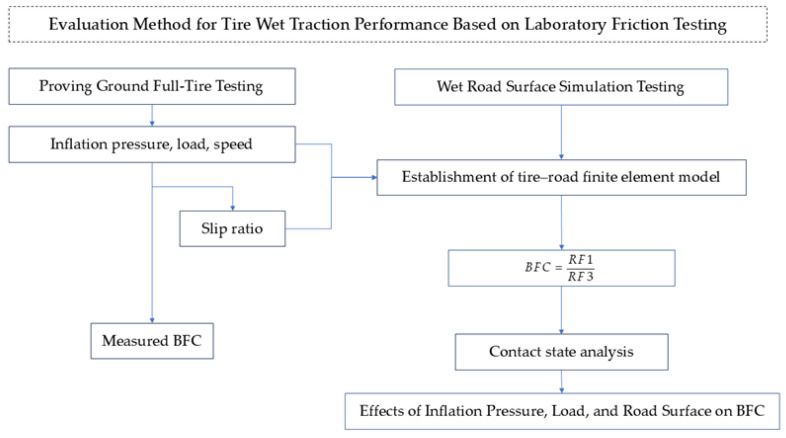
Methodology flowchart.

According to the European Union tire labeling regulation ECE-R117 [25], the BFC represents the ratio of braking force to vertical load. This measure is currently acknowledged as a crucial performance indicator for wet grip in tires. This study introduces a methodology for calculating the tire braking force coefficient by integrating laboratory tests on sliding friction across simulated wet surfaces with computer simulations of tire braking on wet roads, as demonstrated in Figure 1.

### 2.2. Experiment

#### 2.2.1. Wet Grip Test for Tread Blocks

**Figure 2:**
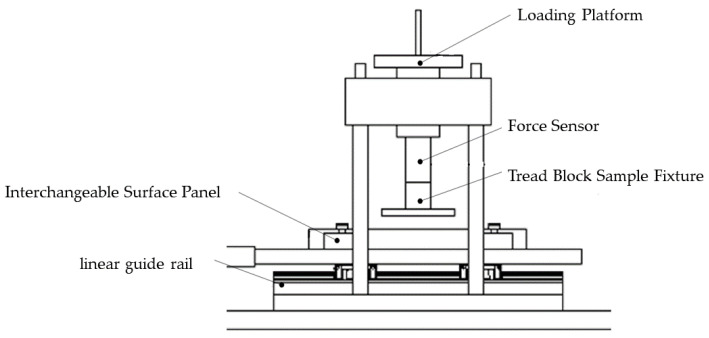
Friction coefficient testing device.

The friction coefficient testing apparatus developed for this study is depicted in Figure 2, showcasing the comprehensive system design. The device comprises four primary components: a normal load mechanism, a horizontal feed system, a tangential force measurement unit, and multiple sample fixtures. The operation involves securely fastening the tire tread pattern sample within the fixture, which then undergoes frictional deformation against an interchangeable road panel while precise normal loads are applied by the loading platform. The normal force exerted by the loading platform is accurately measured using a force transducer. An AC servo motor drives the horizontal motion stage, facilitating controlled reciprocating movement of the interchangeable road panel at specified velocities. A dedicated tangential force measurement unit, positioned between the road panel and motion platform, continually monitors the lateral forces acting on the tread sample. Following Coulomb’s friction law, the system calculates the dynamic friction coefficient of the tread sample by correlating the measured tangential and normal forces.

**Figure 3:**
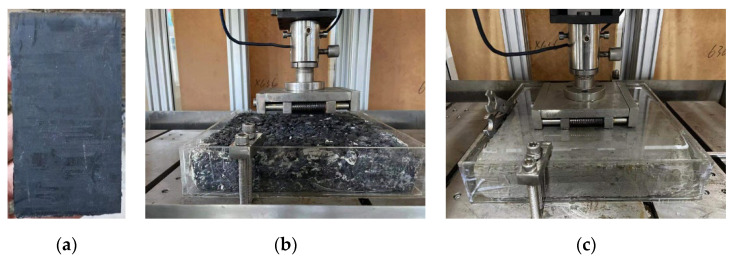
(**a**) Rubber block specimens; (**b**) asphalt pavement surface; (**c**) concrete pavement surface.

Rectangular rubber block specimens, measuring 3 mm × 12 mm × 252 mm, were prepared as illustrated in Figure 3a. As shown in Figure 3b,c, asphalt and concrete pavement surfaces were individually submerged in water tanks to ensure a consistent water film thickness of 2 mm. Friction coefficient measurements were taken under four distinct surface conditions: dry asphalt, wet asphalt, dry concrete, and wet concrete. All experiments were performed under controlled conditions. Specifically, the experiment was conducted at room temperature. The procedure was as follows: first, a stable normal load of 210 N was applied to the specimen; second, the specimen was driven to slide over a total distance of 20 mm at a constant speed of 100 mm/min; finally, to ensure the reliability of the data, three replicate experiments were performed for each group of tread blocks, and the test results were derived exclusively from the linear region of the data curve.

#### 2.2.2. Tire Wet Grip Test

**Figure 4:**
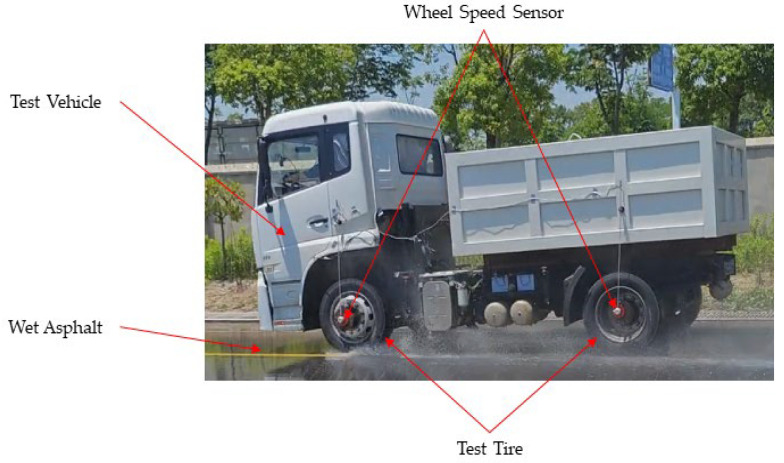
Tire wet grip test.

In accordance with the ECE-R117 test protocol, the testing conditions require an ambient temperature of 5 to 35 degrees Celsius; the test surface is meticulously prepared to ensure a maximum slope of 2% and a uniform surface texture. A water film, maintained at a thickness of 2 mm, is applied using dual spray systems strategically positioned along both sides of the test track. The test tires are fitted onto a test vehicle equipped with an ABS. After accelerating to a speed of 65 km/h, the driver applies full braking until the vehicle comes to a complete stop, with the braking distance documented by the test instrumentation, as illustrated in Figure 4. Each tire configuration undergoes a minimum of six braking trials. The wet braking performance is evaluated by measuring the average deceleration within the speed range of 60 km/h to 20 km/h.

In high-speed emergency braking situations, the ABS adjusts tire rotation to maintain an optimal slip ratio, ensuring effective stopping power. For testing wet grip performance, this slip ratio serves as a crucial parameter that reflects the tire’s braking condition. Mathematically, the slip ratio is defined by Equation (1) as the normalized difference between the vehicle’s longitudinal velocity and the product of the wheel’s angular velocity and effective rolling radius.

(1) $$S=\frac{v-r\omega }{v}\times 100\%$$

where S is the slip ratio, r is the tire’s rolling radius, v is the sliding velocity, and ω is the wheel’s angular velocity.

To accurately determine the dynamic slip ratio between the vehicle’s speed and wheel speed during braking tests, high-resolution wheel speed sensors were installed on both the front and rear axles of the test vehicle. As illustrated in Figure 4, these speed sensors provided synchronized measurements of tire rotational velocity and vehicle longitudinal speed throughout wet braking events, allowing for precise calculations of transient slip conditions.

#### 2.2.3. Finite Element Model of Tire

**Figure 5:**
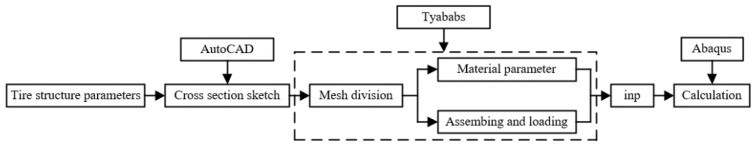
Modeling flowchart.

This study employed a dual-software approach for the finite element modeling of tires, utilizing both the commercial package ABAQUS and the specialized tire analysis software TYABAS v3.0 [26]. Developed by the authors’ research laboratory, TYABAS v3.0 facilitated advanced tire-specific simulations. The complete modeling process is illustrated in Figure 5.

**Figure 6:**
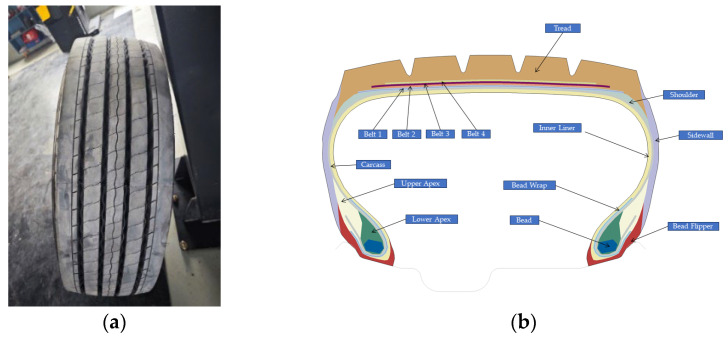
(**a**) The tire; (**b**) diagram of the tire material distribution.

A 295/60R22.5 all-steel radial tire was selected as the research object in this study, as illustrated in Figure 6a. This tire has a complex composite structure, consisting of rubber components like the tread, sidewall, apex, and inner liner, along with reinforcing components such as the belt, carcass, and bead. Figure 6b presents the material distribution of this tire, where different colors correspond to distinct materials.

**Figure 7:**
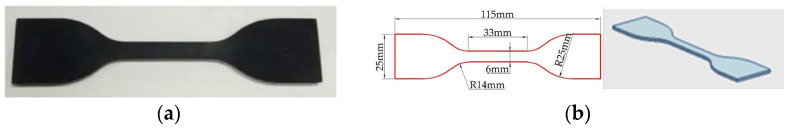
(**a**) Dumbbell-shaped specimen; (**b**) specimen dimensions.

**Figure 8:**
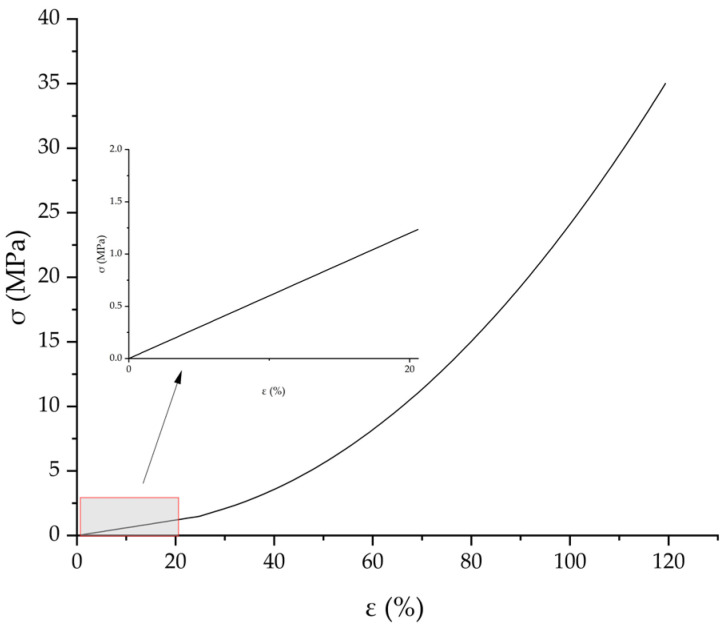
Stress–strain curve.

Tire tread rubber composites were used to prepare the test specimens, which conformed to the specifications of GB/T 528—2009 [27]. Specifically, the specimens were dumbbell-shaped Type I with a thickness of 2 mm, a width of 6 mm, and a gauge length of 25 mm, as illustrated in Figure 7. Tensile tests were conducted in accordance with GB/T 528—2009 using a computer-controlled electronic universal testing machine: The tests were performed at a speed of 500 mm/min under normal temperature conditions with the aim of obtaining the tensile curves of the tread rubber composites. A total of five sets of parallel tests were carried out; after averaging the test data, the constitutive curve of the tread rubber composites was derived, as presented in Figure 8. Within a strain range not exceeding 20%, the constitutive relationship of the tread rubber composites exhibits a linear elastic trend, where stress and strain approximately satisfy a linear correlation.

**Figure 9:**
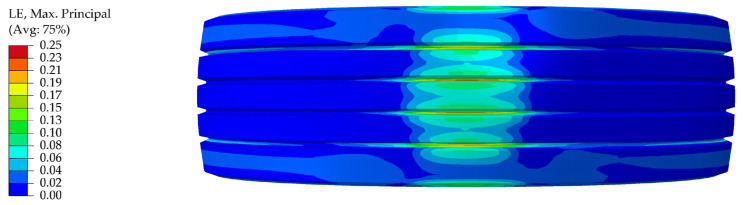
Static contact patch tread strain distribution of tire.

Additionally, static tire model tests indicate that, due to the high rigidity of the reinforcing components, strains within the tire’s rubber matrix rarely exceed 20%, as presented in Figure 9. Consequently, a linear elastic approximation method that accounts for large displacements yields tire mechanical behavior nearly identical to that of the non-linear material model while significantly reducing solution times [28,29]. This linear elastic approximation has no impact on the calculated value of BFC.

**Figure 10:**
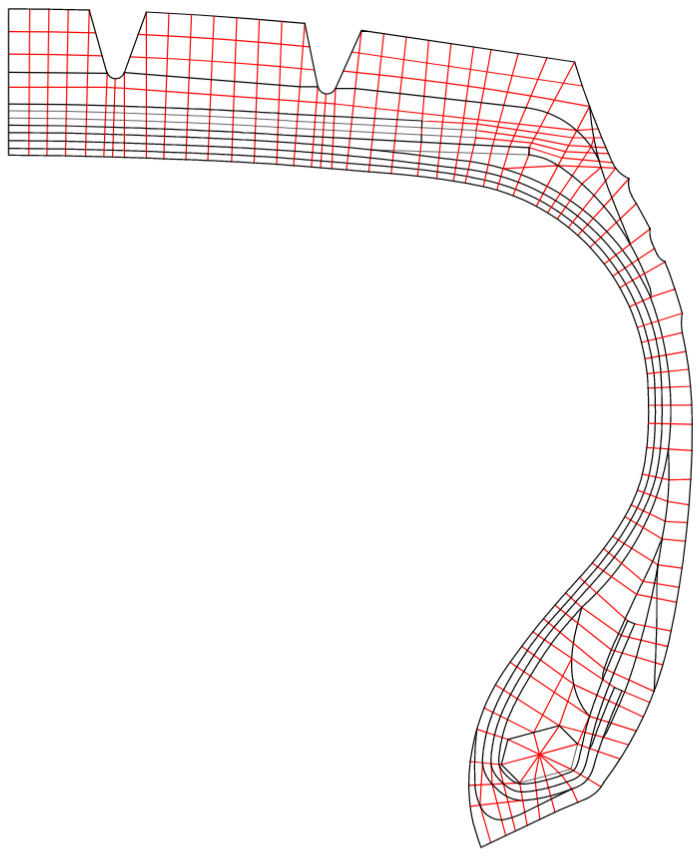
Diagram of the 2D finite element mesh of the tire. Black lines delineated component boundaries and red lines indicated the finite element mesh.

The modeling process commenced with the creation of a two-dimensional CAD model depicting the tire’s material layout. Leveraging the tire’s symmetry, only a half-section was modeled, where black lines delineated component boundaries and red lines indicated the finite element mesh, as illustrated in Figure 10. This model was subsequently imported into TYABAS v3.0 software for preprocessing, which included tasks such as mesh generation, material property assignment, rim configuration, and skeleton element creation. For the 2D finite element model, the cord-rubber composite was simulated using the reinforced membrane model in ABAQUS. The rubber matrix was discretized using CGAX4H elements, while the reinforcing cords were represented as Rebar elements. The material parameters for each component are detailed in Table 1. The tire’s center point was selected as the reference location to facilitate the application of boundary conditions. The rim cross-section was defined according to specific rim dimensions to establish constraints and contact interactions between the rim and tire.

**Figure 11:**
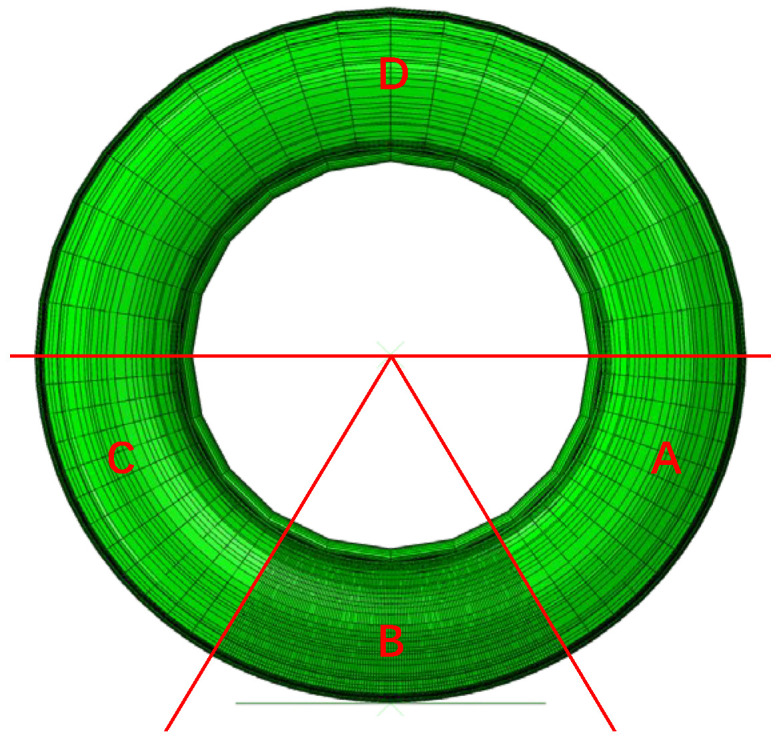
3D finite element model of the tire. Zone B is tire–road contact area while Zones A, C, and D are not in contact with the road.

Following the successful creation of the 2D finite element model, a restart analysis was conducted to transfer data for the development of the 3D finite element model. Leveraging the preprocessing from the 2D model, the SYMMETRIC MODEL GENERATION command was employed to revolve the model 360° around the central axis, resulting in a complete 3D finite element representation of the tire. An analytical rigid surface was incorporated to simulate the road, with the central contact point between the tire and the surface designated as the reference point. The generation of the 3D model was achieved through axisymmetric revolution. For numerical analysis purposes, the tire geometry was segmented into 164 circumferential cross-sections, with the first section aligned along the horizontal axis of Zone A. Special emphasis was placed on Zone B, which was divided into 120 cross-sections to provide a detailed representation of the tire–ground contact mechanics, as illustrated in Figure 11.

**Figure 12:**
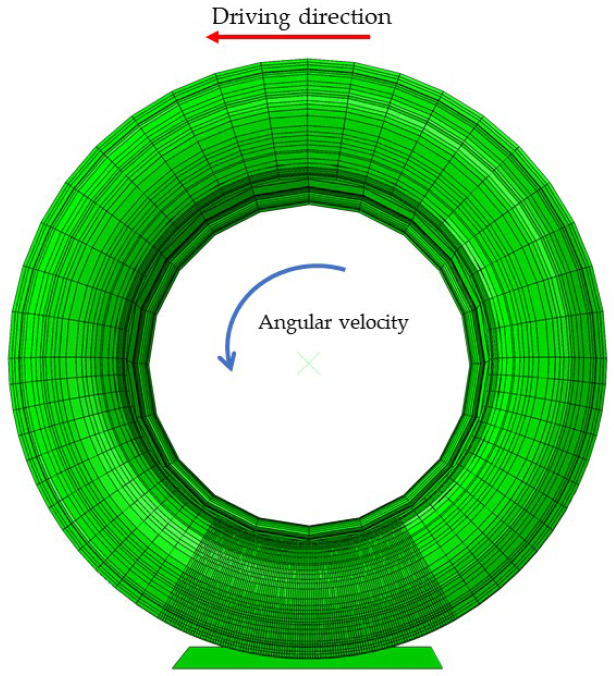
Braking simulation model of the tire.

This study employs the principle of “equivalent discrete braking interval” as developed by the Korean scholar J.R. Cho [17]. Numerical analyses were conducted under various velocity conditions, beginning at 65 km/h during the constant-speed phase, followed by braking at 60 km/h, 40 km/h, and 20 km/h. To accurately simulate ABS braking dynamics, the tire’s rotational angular velocity was meticulously controlled to attain the specified slip ratio conditions. Friction coefficients were assigned based on laboratory measurements of tread rubber in contact with different road surfaces, thereby ensuring a realistic representation of diverse road conditions. The final tire model for wet-road braking analysis is illustrated in Figure 12.

After completing the calculation, *RF*1 and *RF*3 were extracted from the road surface reference point in the simulation results. The simulation calculation of *BFC* is shown in Equation (2).

(2) $$BFC=\frac{RF1}{RF3}$$

where *RF*1 represents the simulated tire braking force and *RF*3 represents the simulated vertical load.

## 3. Results and Discussion

### 3.1. Wet Grip Performance Evaluation

**Figure 13:**
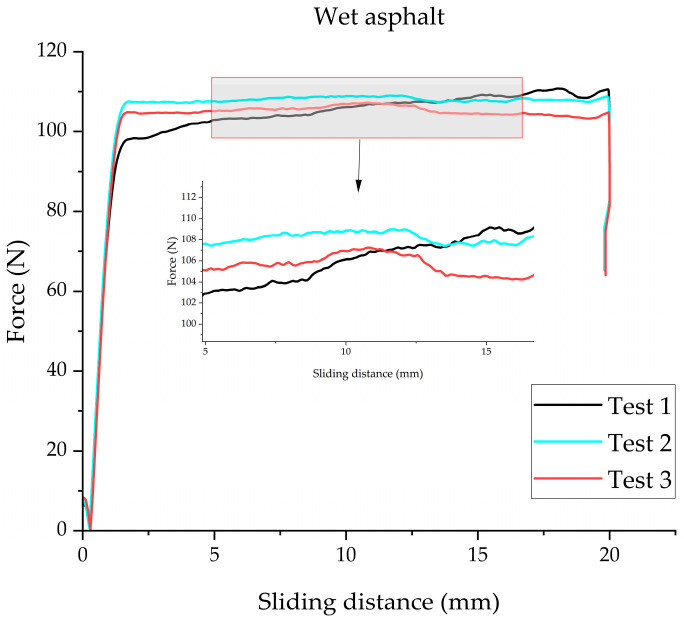
Tangential force test results of tread rubber samples on wet asphalt surface.

To evaluate the effectiveness of the proposed wet grip performance simulation methodology, a thorough comparison was conducted between the finite element simulation results and the experimental data of test tires. The tangential force of tread rubber samples tested on a wet asphalt surface is presented in Figure 13. To determine the tangential force borne by the samples, the stable linear region during the sliding process was selected for analysis; furthermore, the average value of results from three replicate tests was used as the representative tangential force of the tread rubber samples. The detailed test results are summarized in Table 2.

The testing institution has reported that the British Pendulum Number (BPN) for wet road surfaces at 20 °C is 63. The difference between the above BPN result and the friction test results of laboratory rubber samples is mainly due to the different specifications of the rubber used in the BPN test and the laboratory samples.

**Figure 14:**
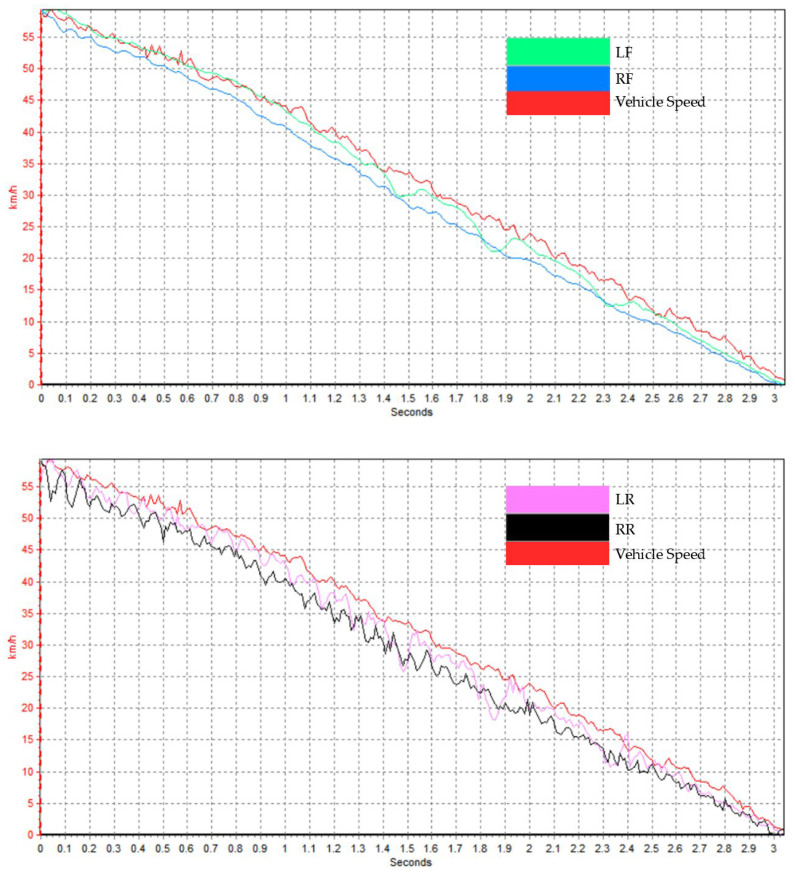
Data was collected from wheel speed and vehicle speed sensors. LF: left front tire; RF: right front tire; LR: left rear tire; RR: right rear tire.

In addition, the test data collected by wheel speed and vehicle speed sensors during tire testing is presented in Figure 14. The results indicate that the ABS performed stably throughout the braking process, with a time-weighted average slip ratio of 7.64% under wet road conditions.

**Figure 15:**
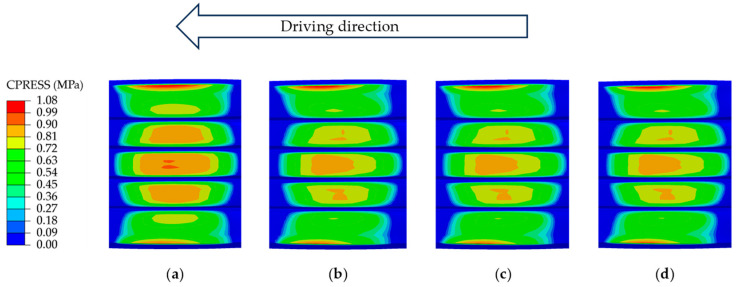
Simulation results of the tire–road contact pressure distribution: (**a**) 65 km/h; (**b**) 60 km/h; (**c**) 40 km/h; (**d**) 20 km/h.

**Figure 16:**
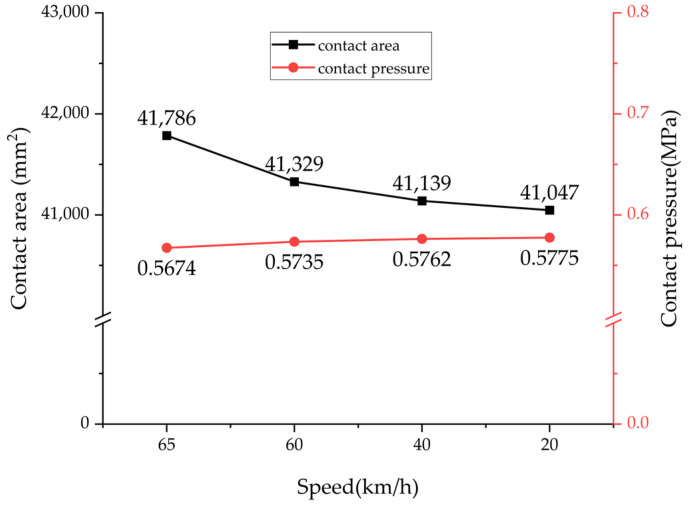
Simulation results of the tire–road contact area and the average contact pressure.

Building on these findings, this study further conducted a finite element simulation of tire wet grip performance using parameters validated with test data. These parameters include an applied load of 2396.5 kg, a tire inflation pressure of 0.63 MPa, and a slip ratio of 7% during braking. The finite element simulation results, presented in Figure 15, clearly reveal distinctly different pressure distribution characteristics within the tire contact patch under the 65 km/h steady-state rolling and braking conditions. When braking is initiated, the tire system undergoes significant longitudinal load transfer, which in turn induces a noticeable redistribution of contact pressure. As shown in Figure 16, during the deceleration process from 65 km/h to 20 km/h, the tire–road contact area decreases by 1.76%, while the contact pressure increases by 1.75%. This variation pattern intuitively reflects the speed dependence of pressure distribution.

**Figure 17:**
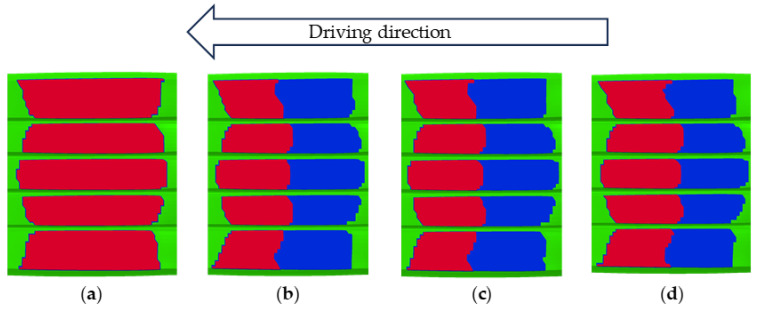
Simulation results of tire–road contact states: (**a**) 65 km/h; (**b**) 60 km/h; (**c**) 40 km/h; (**d**) 20 km/h. Red is the Adhesion zone; Blue is the Slip zone; Green is the No contact zone.

**Figure 18:**
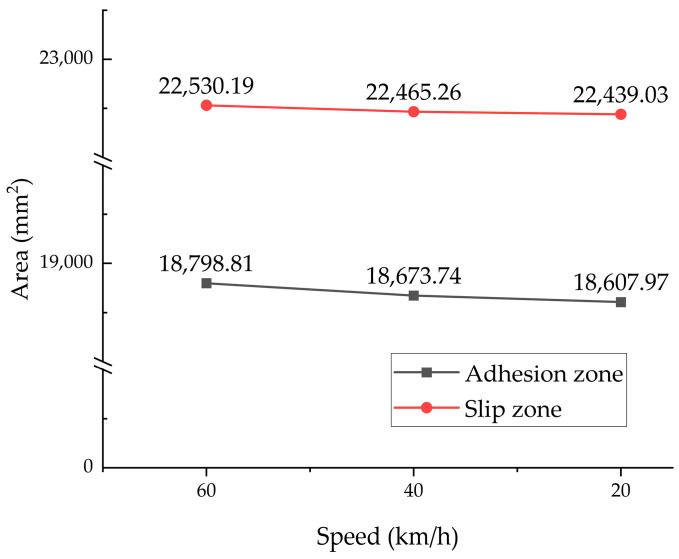
Tire–road contact area simulation results at various braking speeds.

As a typical viscoelastic polymer composite, tire tread rubber exhibits friction behavior at the tire–pavement interface that differs from the traditional Coulomb friction model. Visualization analysis of the tire–road contact state revealed distinct interaction regimes: red zones indicated adhesion, blue highlighted slip, and green represented regions without contact. Under steady-state rolling conditions at 65 km/h, the entire contact patch remained fully adherent to the road surface. In contrast, during braking, a clear distinction emerged between adhesion and slip zones, as illustrated in Figure 17. Notably, as the tire–road contact area decreases with speed, the areas of the adhesion zone and the slip zone also decrease. During braking from 60 to 20 km/h, the adhesion and slip zones decreased by 1.01% and 0.41% respectively, as illustrated in Figure 18.

**Figure 19:**
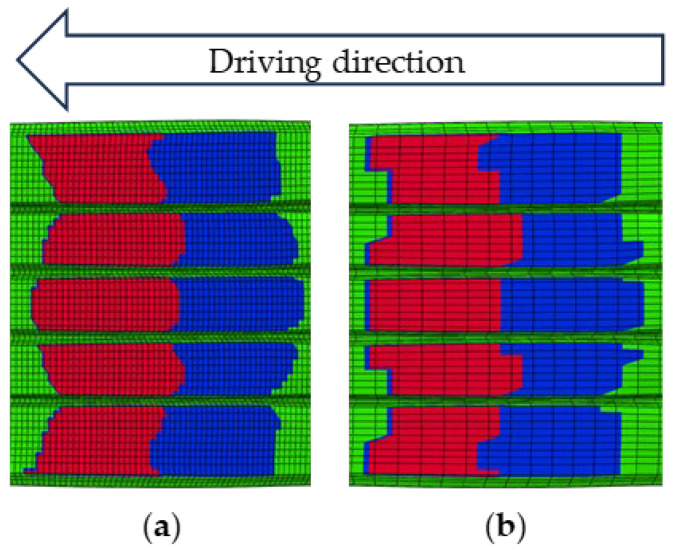
(**a**) 120 cross-sections; (**b**) 30 cross-sections. Red is the Adhesion zone; Blue is the Slip zone; Green is the No contact zone.

It is worth noting that when the contact area is divided into only 30 cross-sections, the boundary between the adhesion zone and the slip zone is relatively poorly defined, making it impossible to accurately identify the spatial distribution of the two zones. As shown in Figure 19, the tire–road contact distributions for the two grid divisions are presented for a braking speed of 20 km/h. Moreover, the contact calculation after reasonable refinement converges well, and the BFC simulation results are stable.

**Figure 20:**
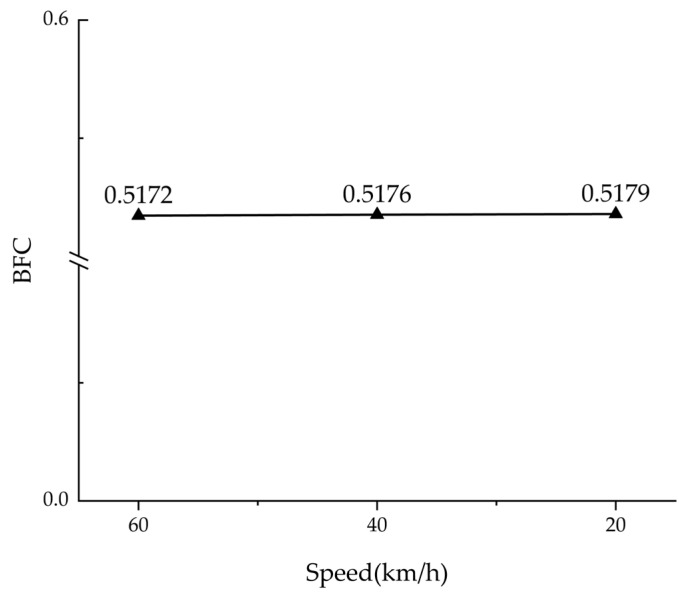
BFC simulation results at various breaking speeds.

Finite element simulation was used to numerically analyze braking friction at three distinct speeds: 20, 40, and 60 km/h. A constant vertical load of 23965 N was applied to the tire model, after which the BFC value was calculated using Equation (2). As illustrated in Figure 20, the simulation results show that the BFC value increases slightly as the speed decreases. Specifically, it increases by 0.07% as the speed decreases from 60 to 20 km/h. By averaging the BFC values during the braking process and rounding the result to two decimal places, the simulated BFC value was determined to be 0.52.

The testing institution reported a test BFC result of 0.56. As shown in Table 3, the relative error between the simulated and test results is 7.14%, thereby highlighting a strong correlation between the numerical model and physical testing. This close alignment not only confirms the reliability of the computational model but also validates the effectiveness of the proposed method in evaluating key parameters influencing tire wet grip performance, particularly under varying braking conditions.

### 3.2. Effect of Road Surface Condition on Tire BFC

**Figure 21:**
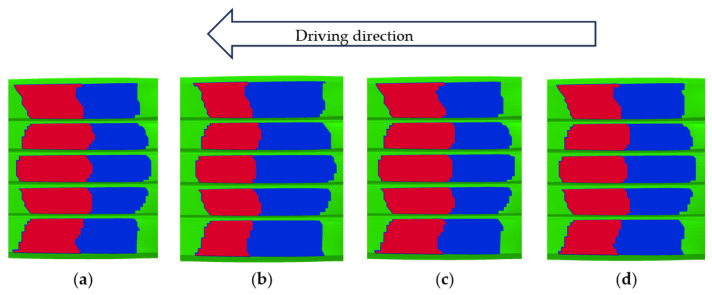
Simulation results of tire–road contact states: (**a**) dry asphalt; (**b**) wet asphalt; (**c**) dry concrete; and (**d**) wet concrete surfaces (water film thickness: 2 mm for wet conditions). Red is the Adhesion zone; Blue is the Slip zone; Green is the No contact zone.

**Figure 22:**
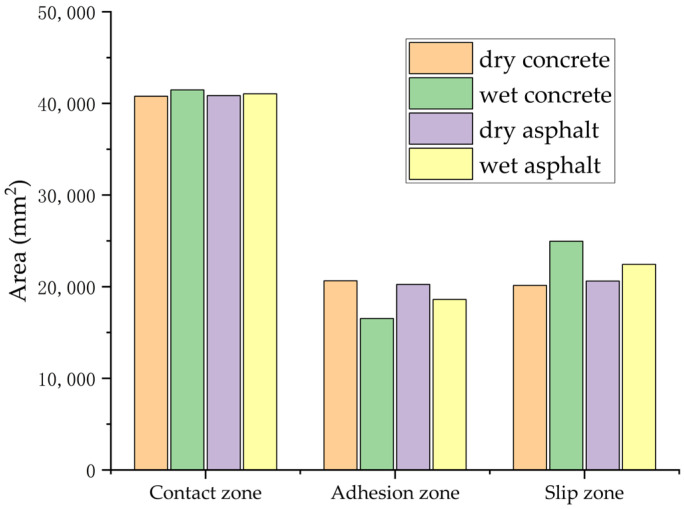
Simulation results of tire–road contact area for four roads.

A comprehensive comparative analysis of tire–road contact states was conducted under four distinct surface conditions: dry asphalt, wet asphalt, dry concrete, and wet concrete, as depicted in Figure 21. The contact mechanics were further clarified through the examination of distributions pertaining to the total contact zones, adhesion zones, and slip zones, as illustrated in Figure 22. The simulation analysis produced systematic measurements of the BFC across all testing conditions, with detailed numerical results provided in Table 4.

A comparative analysis reveals a substantial decrease in the BFC when assessing wet asphalt in contrast to dry conditions. Although the overall tire contact area exhibits a modest increase of 0.47% under wet conditions, significant modifications in contact mechanics are observed: the adhesion area reduces by 8.8%, whereas the slip area enlarges by 8.15%. These interface alterations result in a 15.42% decline in BFC, highlighting the crucial influence of surface wetness on tire–road friction.

On wet concrete pavement, the BFC experiences a substantial decline of 50.39% relative to dry conditions. Quantitative analysis shows that the total tire–concrete contact area slightly increases by 1.67% when wet; however, the changes in contact mechanics are quite pronounced. The adhesion area sees a sharp reduction of 24.93%, while the slip area increases by 19.29%. These significant shifts in contact regions result in the BFC being halved, emphasizing the pronounced influence of surface wetness on tire–concrete friction dynamics.

### 3.3. Effect of Braking Slip Ratio on Tire BFC Under Wet Asphalt Pavement Conditions

**Figure 23:**
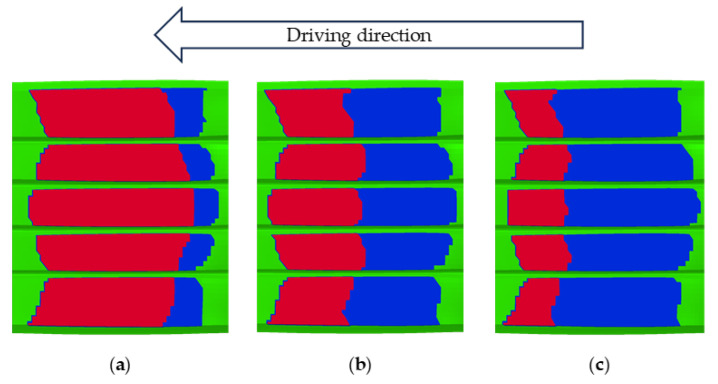
Simulation results of tire–road contact states: (**a**) 4% braking slip ratio; (**b**) 7% braking slip ratio; (**c**) 10% braking slip ratio.

**Figure 24:**
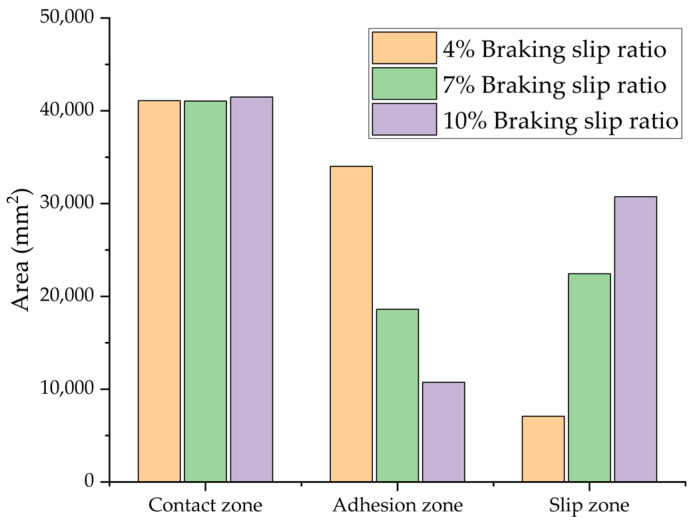
Simulation results of tire–road contact area at different braking slip ratios.

In a controlled laboratory environment simulating wet asphalt pavement, the interfacial characteristics between tires and the road were assessed under consistent inflation pressure and vertical load across varying braking slip ratios. Figure 23 illustrates the changing distributions of the contact state, while Figure 24 presents a quantitative comparison of the total contact area and its components, including adhesion and slip zones. The computationally derived BFC for each test condition is systematically outlined in Table 5.

The simulation results demonstrate a strong positive correlation between the BFC and the slip ratio. Quantitative analysis reveals that as the slip ratio increases from 4% to 7% and subsequently to 10%, there are distinct variations in the contact characteristics. While the overall contact area exhibits only minor fluctuations of 0.11% and 0.96%, the interfacial mechanics undergo significant changes. Notably, the adhesion area decreases by 45.28% and 68.40%, respectively, whereas the slip area increases by 216.52% and 333.63%. These modifications at the interface are associated with notable enhancements in BFC of 29.20% and 44.34%.

### 3.4. Effect of Tire Inflation Pressure on BFC Under Wet Asphalt Pavement Conditions

**Figure 25:**
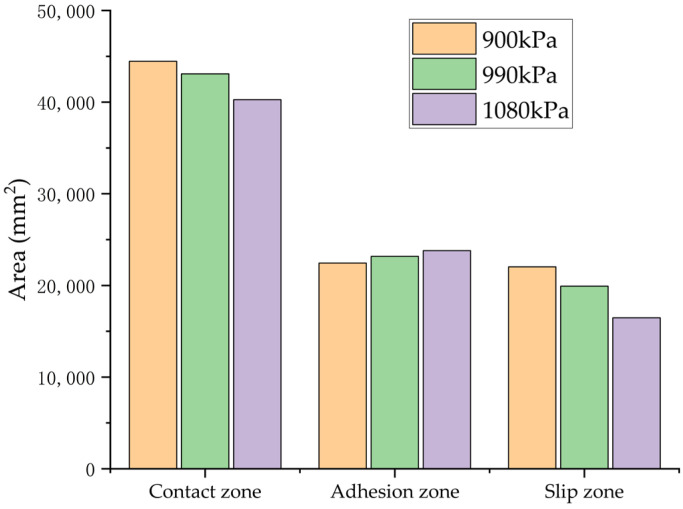
Simulation results of tire–road contact area under different tire inflation pressures.

In a controlled laboratory environment simulating wet asphalt pavement, Figure 25 illustrates the quantitative distribution of tire–road contact characteristics, including total contact area, adhesion area, and slip area, across varying inflation pressures, constant vertical load, and braking slip ratio conditions. The BFCs, derived from computational analysis, are systematically tabulated in Table 6.

The simulation results reveal an inverse relationship between tire inflation pressure and the BFC. When the tire pressure was increased from 900 KPa to 990 KPa and subsequently to 1080 KPa, significant variations in contact mechanics were observed: the total contact area decreased by 3.09% and 9.43%, respectively. Conversely, the area of the adhesion zone experienced increases of 3.28% and 6.10%, while the area of the slip zone was markedly reduced by 9.58% and 25.25%. These alterations in contact characteristics induced by pressure variations resulted in notable reductions in the BFC of 2.8% and 5.87%, correspondingly.

### 3.5. Effect of Tire Vertical Load on BFC Under Wet Asphalt Pavement Conditions

**Figure 26:**
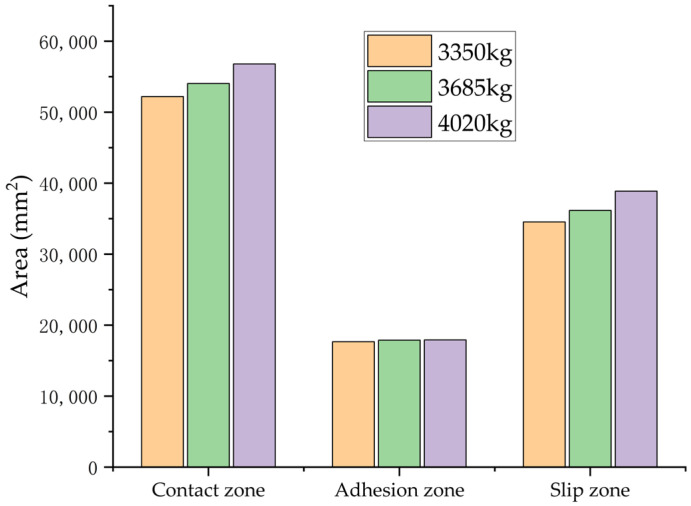
Simulation results of tire–road contact area under different loads.

In a controlled laboratory environment simulating wet asphalt pavement, Figure 26 demonstrates the quantitative distribution of tire–road contact characteristics, including total contact area, adhesion area, and slip area, across different vertical loads, constant inflation pressure, and braking slip ratio conditions. The BFCs, obtained through computational analysis, are systematically presented in Table 7.

The simulation results indicate that variations in vertical load have minimal impact on the BFC. When the load increased from 3350 kg to 3685 kg and then to 4020 kg, there were observable changes in contact mechanics: the total contact area expanded significantly by 3.53% and 8.81%, respectively. Similarly, the adhesion area experienced a slight increase of 1.22% and 1.46%, while the slip area decreased by 4.71% and 12.57%. Importantly, these alterations in contact properties due to changes in load did not result in statistically significant differences in the BFC.

## 4. Conclusions

Tire treads are fabricated using rubber polymers as the matrix material. During the tire braking process, the tire–road contact area is divided into adhesion and slip zones, rendering the traditional Coulomb friction model unsuitable. This study focuses on the testing conditions for the wet skid performance of all-steel radial tires. Specifically, the test was conducted at 5–35 °C, the test road surface was paved with a 2 mm thick water film, and the discussion centers on the BFC values within the 60–20 km/h braking speed range with the ABS activated.

This study compared the BFC performance of the tire on dry and wet asphalt, as well as dry and wet concrete surfaces. Under wet conditions, the adhesion area decreased sharply, while the slip area expanded significantly; these changes led to a notable reduction in BFC, especially on wet concrete surfaces. Further analysis investigated the effects of braking slip ratio, inflation pressure, and vertical load on BFC. The simulation results indicated that BFC increased with the rise in braking slip ratio, decreased slightly with the increase in tire inflation pressure, and exhibited relatively low sensitivity to vertical load variations.

The proposed model exhibits excellent performance under various driving conditions and offers three key advantages: strong predictive ability, good consistency with experimental data, and accurate simulation of contact area evolution. This method has the potential to optimize tire structural design and provide targeted guidance for the formulation optimization of tire tread rubber, thereby improving tire wet skid performance.

Based on the research conducted in this paper, in future work, we will systematically incorporate more influencing factors and expand the scope of application of this research method, ultimately providing theoretical and practical references for more challenging methodological application scenarios in the future.

## Figures and Tables

**Table 1:** Material parameters used in the simulation.

Material	Young’s Modulus (MPa)	Poisson’s Ratio
Upper Apex	5.95 × 10^0^	0.49
Lower Apex	1.85 × 10^1^	0.49
Inner Liner	2.58 × 10^0^	0.49
Bead Flipper	1.36 × 10^1^	0.49
Bead Wrap	8.42 × 10^4^	0.30
Belt 1	1.21 × 10^5^	0.30
Belt 2	1.21 × 10^5^	0.30
Belt 3	1.21 × 10^5^	0.30
Belt 4	1.21 × 10^5^	0.30
Bead	1.80 × 10^5^	0.30
Tread	5.96 × 10^0^	0.49
Sidewall	3.74 × 10^0^	0.49
Shoulder	3.73 × 10^0^	0.49
Carcass	1.48 × 10^5^	0.30

**Table 2:** Tangential force test results of tread rubber samples.

Surface	Tangential Force (N)
dry concrete	145.02
wet concrete	86.13
dry asphalt	134.42
wet asphalt	108.72

**Table 3:** Comparison between BFC simulated and test values.

	Simulated Result	Test Result
BFC	0.52	0.56

**Table 4:** BFC simulation results at the four road surfaces.

Surface	BFC
dry concrete	0.54
wet concrete	0.36
dry asphalt	0.52
wet asphalt	0.45

**Table 5:** BFC simulation results at different braking slip ratios.

Braking Slip Ratio	BFC
4%	0.35
7%	0.45
10%	0.50

**Table 6:** BFC simulation results under different tire inflation pressures.

Pressure (kPa)	BFC
900	0.42
990	0.41
1080	0.40

**Table 7:** BFC simulation results under various load conditions.

Load (kg)	BFC
3350	0.45
3685	0.45
4020	0.45

## Data Availability

The original contributions presented in this study are included in the article. Further inquiries can be directed to the corresponding author.
